# Phytochemical Profiling and Anti-Inflammatory Activity of *Rubus parvifolius* Leaf Extract in an Atopic Dermatitis Model

**DOI:** 10.3390/life15091383

**Published:** 2025-09-01

**Authors:** Junseong Kim, Derrick Kakooza, Chang-Dae Lee, Su-Young Jung, Kyung Choi, Sung-Kwon Moon, Hoon Kim, Sanghyun Lee

**Affiliations:** 1 Department of Plant Science and Technology, Chung-Ang University, Anseong 17546, Republic of Korea; junni1227@naver.com (J.K.); great7321@naver.com (C.-D.L.); 2 Department of Food and Nutrition, Chung-Ang University, Anseong 17546, Republic of Korea; kakoda9403@cau.ac.kr (D.K.); sumoon66@cau.ac.kr (S.-K.M.); 3 Forest Biodiversity Research Division, Korea National Arboretum, Pocheon 11186, Republic of Korea; neverland81@korea.kr (S.-Y.J.); kchoi69@korea.kr (K.C.); 4 Department of Food and Nutrition, Anyang University, Anyang 14028, Republic of Korea; 5 Natural Product Institute of Science and Technology, Anseong 17546, Republic of Korea

**Keywords:** *Rubus parvifolius*, atopic dermatitis, LC-MS/MS, HPLC, anti-inflammatory, HaCaT cells, plant-based therapy

## Abstract

This study investigated the anti-inflammatory effects of *Rubus parvifolius* leaf (RPL) extract, and its phytochemical composition was characterized using LC-MS/MS and HPLC analyses. In an inflammation model using HaCaT keratinocytes, treatment with RPL extract led to a significant reduction in inflammatory markers, indicating strong anti-inflammatory activity. Phytochemical analysis revealed that the extract is rich in flavonoids, with quercetin 3,7-diglucoside (12.73 mg/g DW) as the most abundant compound, followed by hirsutrin (4.74 mg/g DW) and ellagic acid (1.58 mg/g DW). Kaempferol 3-*O*-glucuronide was detected in lower amounts (0.31 mg/g DW), and tiliroside was present only in trace levels. These compounds are well known for their antioxidant and anti-inflammatory properties, suggesting that RPL extract may exert multiple beneficial effects on skin health. Collectively, these findings support the potential of RPL extract as a natural therapeutic agent for managing skin inflammation, particularly in conditions such as atopic dermatitis, with its efficacy likely attributed to the high levels of quercetin 3,7-diglucoside, hirsutrin, and ellagic acid. However, the present work was confined to in vitro experiments, and the mechanistic pathways were not experimentally validated. Future in vivo studies are needed to confirm these findings.

## 1. Introduction

Atopic dermatitis (AD) is a common chronic inflammatory skin disorder affecting both children and adults. It is associated with immune dysregulation, epidermal barrier dysfunction, and elevated pro-inflammatory cytokines, including interleukin-6 (IL-6), interleukin-8 (IL-8), and monocyte chemoattractant protein-1 (MCP-1) [1]. These cytokines promote immune cell infiltration, leading to sustained inflammation and typical symptoms such as erythema, pruritus, and skin thickening. Topical corticosteroids and systemic immunosuppressants are the main treatments for AD. However, their long-term use is limited by side effects, including skin atrophy, delayed wound healing, and increased risk of infections [2]. Consequently, there is growing interest in safer, plant-based therapeutics that can manage inflammation while minimizing adverse effects [3].

Several plant-derived treatments have shown potential to alleviate AD symptoms. Chelidonic acid reduces itching and skin redness, lowers IgE levels, and suppresses inflammatory markers in both cell and animal models [4]. Paedoksan, a traditional multi-herb formulation, inhibits allergic cell activation and blocks STAT6 signaling, a pathway associated with IL-4-driven inflammation [5]. Additionally, an ethanolic extract of *Artemisia apiacea* decreases inflammation-related cytokine production in immune-stimulated skin cells and improves skin lesions in a mouse model of AD [6].

*Rubus parvifolius* L. is a deciduous shrub native to East Asia and Oceania. It is being explored for therapeutic potential, but studies on its efficacy are still scarce, particularly for inflammatory skin diseases. Although no studies have directly assessed its effects in AD models, *R. parvifolius* extracts exhibit antioxidant and anti-melanogenic activities in cultured skin cells under oxidative stress [7]. Traditionally, *R. parvifolius* has been used in East Asia to treat inflammation-related conditions, including skin disorders, providing an ethnopharmacological rationale for its investigation [8]. Moreover, research on other *Rubus* species has demonstrated that leaf extracts rich in flavonoids can modulate inflammatory pathways relevant to AD, supporting the choice of *R. parvifolius* as a candidate for anti-inflammatory and dermatological studies [9].

Phytochemical analyses of *Rubus* species have identified several flavonoids, tannins, and triterpenoids [10]. Quercetin can downregulate pro-inflammatory cytokines such as IL-6, IL-8, and MCP-1 via NF-κB pathway inhibition [11]. Kaempferol and rutin scavenge reactive oxygen species (ROS) and support epithelial barrier function, while ellagic acid protects against UV-induced skin damage [12,13]. Despite these findings, the use of *R. parvifolius* leaf to treat inflammatory skin conditions such as AD remains unexplored.

This study sought to address this gap by (1) evaluating the anti-inflammatory effects of *R. parvifolius* leaf extract in a tumor necrosis factor-α (TNF-α) and interferon-γ (IFN-γ)-stimulated HaCaT keratinocyte model, and (2) performing liquid chromatography–tandem mass spectrometry (LC-MS/MS) and high-performance liquid chromatography (HPLC) analyses to identify major flavonoids in ethanol extracts of *R. parvifolius* leaf. To our knowledge, this is the first study to investigate the dermatological potential of *R. parvifolius* in an AD-relevant context, combining both chemical profiling and biological evaluation to support its potential as a plant-derived therapeutic for chronic inflammatory skin disorders. These findings provide new insights into plant-derived therapeutics for chronic inflammatory skin disorders and may guide future research into natural anti-inflammatory agents.

## 2. Materials and Methods

### 2.1. Plant Materials and Sample Preparation

*R. parvifolius* leaves (RPL) were collected from Mt. Bukhansan, Yangju, Republic of Korea. A voucher specimen (K. Choi s.n., 20241123) was deposited at the Korea National Herbarium of the Korea National Arboretum in Pocheon, Republic of Korea. The sample was air-dried indoors, and ground using a blender. RPL (10 g) was extracted with 300 mL of 95% ethanol (30-fold volume) under reflux conditions for 3 h. This extraction process was repeated three times to ensure maximal recovery of phytochemicals. After extraction, the combined solutions were filtered to remove solids and concentrated under reduced pressure to obtain the crude extract.

### 2.2. Chemicals

**Figure 1:**
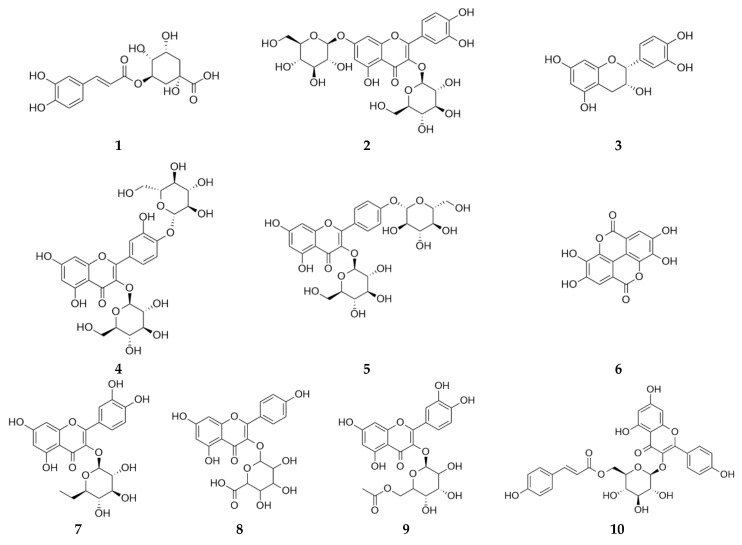
Chemical structures of chlorogenic acid (**1**), quercetin 3,7-diglucoside (**2**), epicatechin (**3**), quercetin 3,4′-diglucoside (**4**), kaempferol 3,4′-di-*O*-glucoside (**5**), ellagic acid (**6**), hirsutrin (**7**), kaempferol 3-*O*-glucuronide (**8**), quercetin-3-glucose-6″-acetate (**9**), and tiliroside (**10**).

Dulbecco’s modified Eagle’s medium (DMEM), 3-(4,5-dimethylthiazol-2-yl)-2,5-diphenyltetrazolium bromide (MTT), and dexamethasone were purchased from Sigma-Aldrich (St. Louis, MO, USA). Recombinant human tumor necrosis factor-alpha (TNF-α; Cat. No. 210-TA-100) and interferon-gamma (IFN-γ; Cat. No. 285-IF-100) were obtained from R&D Systems (Minneapolis, MN, USA). Fetal bovine serum (FBS; Cat. No. 35-015-CV) and penicillin-streptomycin (P/S; Cat. No. 30-002-CI) were purchased from Corning Life Sciences (Manassas, VA, USA). Ethanol (95%) and HPLC-grade dimethyl sulfoxide (DMSO) were purchased from Samchun Chemicals (Pyeongtaek, Republic of Korea). HPLC-grade methanol was obtained from Sigma-Aldrich (Burlington, MA, USA), while acetonitrile (ACN) and ultrapure water for HPLC were purchased from Honeywell (Burdick and Jackson, Muskegon, MI, USA). Phosphoric acid, used as a mobile phase modifier, was obtained from Fisher Scientific (Loughborough, UK). Chlorogenic acid (**1**), quercetin 3,7-diglucoside (**2**), epicatechin (**3**), quercetin 3,4′-diglucoside (**4**), kaempferol 3,4′-di-*O*-glucoside (**5**), ellagic acid (**6**), hirsutrin (**7**), kaempferol 3-*O*-glucuronide (**8**), quercetin-3-glucose-6″-acetate (**9**), and tiliroside (**10**) were obtained from the Natural Product Institute of Science and Technology (www.nist.re.kr; accessed on 24 March 2025), Anseong, Republic of Korea (Figure 1).

### 2.3. Equipment

Chromatographic separation was carried out using a Waters Alliance e2695 system coupled with a 2998 photodiode array detector (Waters, Milford, MA, USA). LC-MS/MS analysis was performed using a Thermo Scientific Ultimate 3000 UHPLC system (San Jose, CA, USA) connected to a TripleTOF 5600+ mass spectrometer (AB Sciex, Framingham, MA, USA).

### 2.4. Cell Culture for Anti-Inflammatory Evaluation

The anti-inflammatory activity of RPL extract was evaluated using HaCaT cells (CLS Cell Line Service, Eppelheim, Heidelberg, Germany). Cells were cultured in DMEM supplemented with 10% heat-inactivated FBS and 1% P/S. The anti-inflammatory evaluation was carried out based on previous studies with minor modifications [14,15]. Briefly, HaCaT cells (passage number 27) were seeded at a density of 1.0 × 10^5^ cells/well in 96-well plates for the MTT assay and enzyme-linked immunosorbent assay (ELISA), and at 2.0 × 10^5^ cells/dish in 60 mm culture dishes (passage number 30) for quantitative reverse transcription PCR (qRT-PCR) analysis. The cells were maintained at 37 °C in a humidified incubator with 5% CO_2_. After stabilization for 24 h, the cells were serum-starved for an additional 24 h in serum-free DMEM containing 1% P/S. The cells were then treated with various concentrations of the RPL extract. For controls, 0.1% DMSO in serum-free DMEM was used as the negative control, and dexamethasone (20 μg/mL) was used as the positive control. Following a 1 h pre-treatment with the extracts or controls, inflammation was induced by stimulating the cells with 10 ng/mL of TNF-α and 10 ng/mL of IFN-γ (TI) added simultaneously to the culture medium. After 24 h of incubation, cell viability was measured using the MTT assay, and absorbance was read at 550 nm with an Epoch microplate spectrophotometer (BioTek Instruments, Winooski, VT, USA). The culture supernatants were collected for cytokine quantification via ELISA. Additionally, total RNA was extracted from the cells for the analysis of inflammatory gene expression using qRT-PCR.

### 2.5. ELISA

Following the 24 h incubation period, cell culture supernatants were collected to evaluate the inhibitory effects of the RPL extract on the secretion of pro-inflammatory cytokines. Cytokine levels were measured using commercial ELISA kits specific for IL-6 (Cat. No. 555240), IL-8 (Cat. No. 555244), and MCP-1 (Cat. No. 555179), all purchased from BD Biosciences (Winooski, VT, USA). HaCaT cells were pretreated with RPL extract (25–100 μg/mL) or dexamethasone (20 μg/mL; positive control, PC) for 1 h, followed by stimulation with TI (10 ng/mL each) for 24 h. Cells treated with 0.1% DMSO in serum-free medium served as the negative control. Cell viability was assessed by the MTT assay, and cytokine concentrations (IL-6, IL-8, and MCP-1) were quantified by ELISA.

### 2.6. qRT-PCR

After 24 h of incubation, TI-stimulated HaCaT cells were washed twice with phosphate-buffered saline (PBS; Dongjin Biotech, Incheon, South Korea). Total RNA was extracted using the NucleoSpin^®^ RNA Plus extraction kit (Macherey-Nagel, Düren, Germany) according to the manufacturer’s instructions. RNA concentrations were normalized using a NanoDrop plate reader and microplate spectrophotometer. Equal amounts of total RNA were then reverse-transcribed into cDNA using the ReverTra Ace™ qPCR RT Master Mix (Toyobo, Osaka, Japan). The resulting cDNA was amplified using SYBR^®^ Green Real-time PCR Master Mix (Toyobo, Osaka, Japan) in combination with specific primers targeting MCP-1, regulated upon activation normal T cell expressed and secreted (RANTES), thymus and activation-regulated chemokine (TARC), macrophage-derived chemokine (MDC), cutaneous T cell-attracting chemokine (CTACK), and IL-6. Glyceraldehyde-3-phosphate dehydrogenase (GAPDH) was used as the endogenous control to normalize gene expression. Relative expression levels were calculated using the 2^−ΔΔCt^ method. The primers used for qRT-PCR analysis are summarized in Table 1. HaCaT cells were pretreated with RPL extract (25–100 μg/mL) or dexamethasone (20 μg/mL; PC) for 1 h, followed by stimulation with TI (10 ng/mL each) for 24 h. Cells treated with 0.1% DMSO in serum-free medium served as the negative control. The expression of pro-inflammatory genes was analyzed using qRT-PCR.

### 2.7. Preparation of Samples and Standard Solutions for HPLC

Sample solutions were prepared at a concentration of 30 mg/mL, and standard solutions at 0.5 mg/mL, using methanol as the primary solvent. DMSO was used as an alternative solvent when needed. All prepared solutions were filtered through polyvinylidene difluoride membrane filters to ensure clarity prior to HPLC analysis.

### 2.8. HPLC/PDA Conditions

Quantitative analysis was conducted using a reverse-phase HPLC system equipped with an INNO C18 column (250 mm × 4.6 mm, 5 µm particle size). The mobile phase consisted of 0.5% aqueous acetic acid (solvent A) and ACN (solvent B), applied in gradient mode. The gradient program was as follows: 0–5 min (5% B), 6 min (10% B), 75 min (28% B), 76–81 min (100% B), and 82–92 min (5% B). The gradient program was initially designed with reference to previously reported methods [16]. However, it was extensively adapted and optimized to improve peak separation, resulting in a longer run time. Repeated analyses confirmed consistent retention times and reproducible peak resolution. While a full validation according to ICH Q2(R1) was not performed, the reproducibility observed during optimization supports the reliability of the method for the present study. Elution was carried out at a flow rate of 1.0 mL/min, with the column temperature maintained at 30 °C. Detection was conducted at a wavelength of 254 nm using a photodiode array detector.

### 2.9. Calibration Curve

Calibration curves were constructed by analyzing each standard at five different concentrations. Linearity was evaluated based on the correlation coefficient (*r*^2^). Compound concentrations in the RPL extract were determined using the corresponding calibration equations. Each curve was generated by plotting peak area (Y) against concentration (X, μg/mL). All quantitative results are expressed as mean ± standard deviation (SD; *n* = 3).

### 2.10. Statistical Analysis

Data are presented as mean ± SD (*n* = 3) from three independent experiments. Statistical comparisons were performed using one-way ANOVA followed by Duncan’s multiple range test, with a *p*-value < 0.05 considered statistically significant. Analyses were conducted using IBM Statistics version 26.0 (IBM Corp., Armonk, NY, USA).

## 3. Results and Discussion

### 3.1. Cell Viability and Inflammatory Response

**Figure 2:**
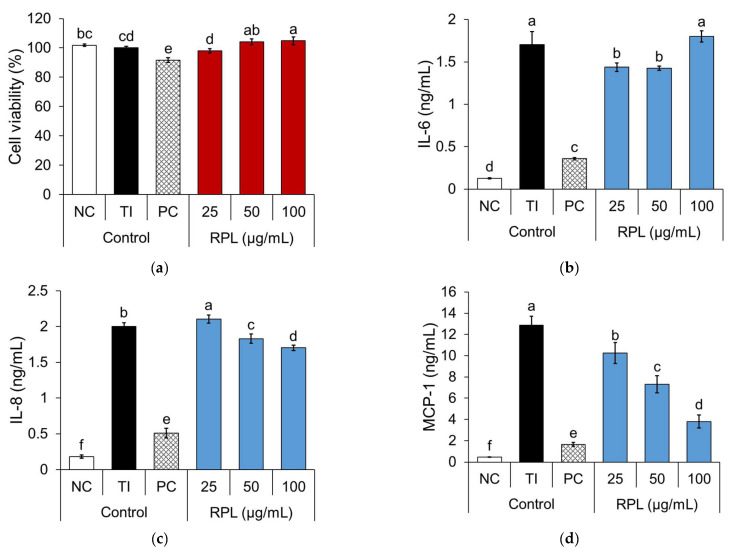
Effects of RPL on cell viability (**a**), and the secretion of IL-6 (**b**), IL-8 (**c**), and MCP-1 (**d**) in TI-stimulated HaCaT cells. HaCaT cells were pretreated with RPL extract and then stimulated with TNF-α and IFN-γ (TI). Cell viability was assessed by MTT assay, and IL-6, IL-8, and MCP-1 levels in the supernatant were determined by ELISA. Data are expressed as mean ± SD (*n* = 3). Statistical significance was evaluated by one-way ANOVA followed by Duncan’s multiple range test; bars with different letters differ significantly (*p* < 0.05).

The cytotoxicity and anti-inflammatory activity of RPL extract were evaluated in TI-stimulated HaCaT cells. MTT assays revealed no cytotoxic effects at concentrations ranging from 25 to 100 µg/mL, with cell viability maintained between 98.0% and 104.8% (Figure 2).

ELISA results demonstrated that TI stimulation significantly increased the secretion of IL-6 (1.7 ng/mL), IL-8 (2.0 ng/mL), and MCP-1 (12.9 ng/mL). As expected, treatment with dexamethasone significantly suppressed the production of these cytokines, reducing IL-6 to 0.5 ng/mL (−81.1%), IL-8 to 0.4 ng/mL (−85.4%), and MCP-1 to 1.7 ng/mL (−90.3%). The RPL extract inhibited IL-6 production at 25 and 50 µg/mL by 17.0% and 17.7%, respectively. RPL with a concentration of 100 µg/mL did not significantly affect IL-6 levels compared with the TI control group and may reflect a non-linear dose-response as reported in other plant-derived bioactive compounds showing a biphasic behavior [17]. IL-8 was reduced at 50 µg/mL (1.8 ng/mL, −9.3%) and 100 µg/mL (1.7 ng/mL, −16.3%), but not at 25 µg/mL. MCP-1 secretion decreased in a dose-dependent manner. Treatment with 25, 50, and 100 µg/mL of RPL extract reduced MCP-1 levels to 10.3, 7.3, and 3.8 ng/mL, respectively, with the highest concentration resulting in 73.0% inhibition. These findings suggest that RPL extract exerts a notable inhibitory effect on the production of pro-inflammatory cytokines IL-6, IL-8, and MCP-1 in the in vitro TI-stimulated AD model.

### 3.2. Inflammatory Gene Expression

**Figure 3:**
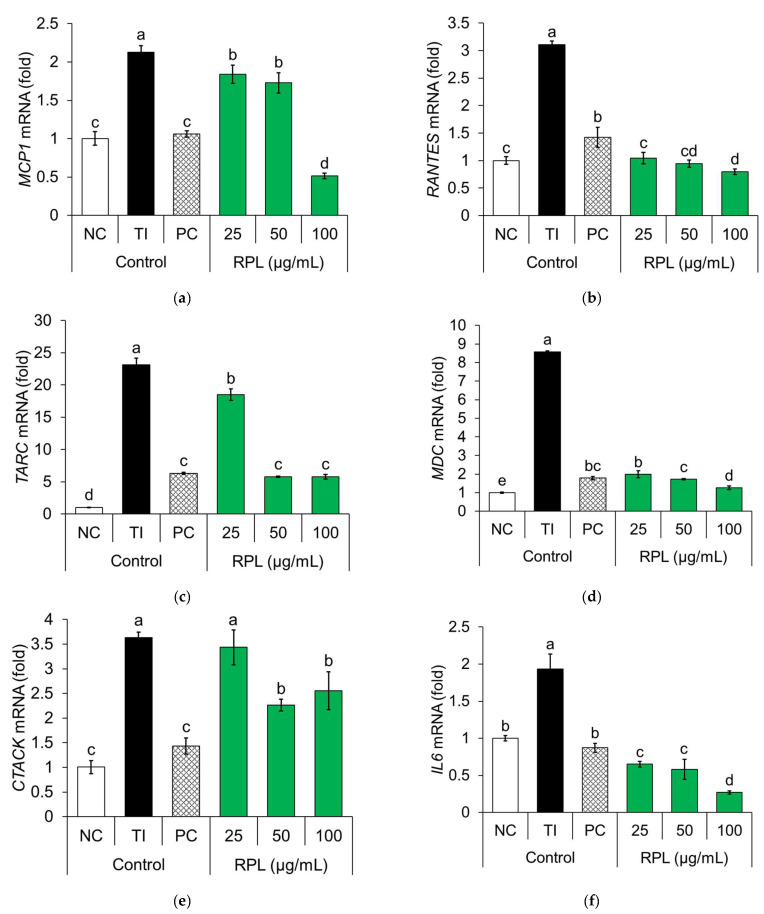
Effects of RPL on pro-inflammatory gene expression in HaCaT cells: MCP-1 (**a**), RANTES (**b**), TARC (**c**), MDC (**d**), CTACK (**e**), and IL-6 (**f**) mRNA expression. HaCaT cells were pretreated with RPL extract (25–100 µg/mL) for 1 h and then stimulated with TNF-α and IFN-γ (10 ng/mL each) for 24 h. mRNA expression of MCP-1, RANTES, TARC, MDC, CTACK, and IL-6 was quantified by qRT-PCR, normalized to GAPDH. Data are expressed as mean ± SD (*n* = 3). Statistical analysis was performed using one-way ANOVA followed by Duncan’s multiple range test. Different letters above the bars indicate significant differences between groups (*p* < 0.05).

The mRNA expression levels of key pro-inflammatory genes (MCP-1, MDC, CTACK, RANTES, TARC, and IL-6) were evaluated in TI-stimulated HaCaT cells using qRT-PCR (Figure 3). TI stimulation significantly upregulated the expression of MCP-1 (2.13-fold), RANTES (3.11-fold), TARC (23.12-fold), MDC (8.58-fold), CTACK (3.63-fold), and IL-6 (3.63-fold) compared to the negative control group. Dexamethasone, used as the PC, markedly suppressed these elevations, reducing gene expression to 1.06-fold (MCP-1), 1.42-fold (RANTES), 6.30-fold (TARC), 1.78-fold (MDC), 1.43-fold (CTACK), and 0.87-fold (IL-6).

Treatment with RPL extract dose-dependently inhibited the expression of all target genes. Specifically, expression was reduced in MCP-1 (1.84–0.51-fold), RANTES (1.05–0.80-fold), TARC (18.50–5.77-fold), MDC (1.99–1.26-fold), CTACK (3.44–2.55-fold), and IL-6 (0.65–0.27-fold) across increasing concentrations. Notably, RPL extract suppressed the expression of RANTES, MDC, and IL-6 more effectively than dexamethasone at comparable concentrations, indicating a potentially greater anti-inflammatory effect. These results highlight the potential of RPL extract as a natural therapeutic agent for alleviating inflammatory skin conditions such as AD.

The anti-inflammatory activity of RPL extract was evaluated using TI-stimulated HaCaT cells, a widely accepted in vitro model for mimicking the inflammatory environment of AD. Cytokine stimulation causes keratinocytes to release chemokines: MCP-1, RANTES, IL-8, TARC, MDC, and CTACK. These chemokines recruit Th2 lymphocytes and other immune cells, aggravating AD pathogenesis [1,18]. In this study, treatment with RPL extract significantly suppressed the expression of these inflammatory genes (MCP-1, RANTES, IL-8, TARC, MDC, and CTACK), as well as the secretion of key pro-inflammatory cytokines, including IL-6, IL-8, and MCP-1, in a dose-dependent manner. Collectively, these findings highlight the ability of RPL extract to downregulate chemokine gene expression and cytokine release, supporting its therapeutic potential as a natural anti-inflammatory agent for managing AD-associated skin inflammation. Despite the promising anti-inflammatory effects observed in this study, several limitations should be acknowledged. First, the experiments were conducted exclusively in an in vitro HaCaT keratinocyte model, which does not fully capture the complex immune interactions and skin environment present in AD patients. Therefore, the current findings should be interpreted with caution, and in vivo studies using murine AD models are necessary to validate the therapeutic potential and safety of RPL extract in a more physiologically relevant context.

### 3.3. LC-MS and HPLC Analysis

Various species within the *Rubus* genus have been studied for their health-promoting properties, with most research focusing on the fruits. For example, the fruits of *Rubus coreanus* are known to contain bioactive constituents such as phenolic compounds and flavonoids, which exhibit antioxidant and anti-inflammatory activities [19]. However, a comparative HPLC-based analysis of four Korean *Rubus* species, including *R. parvifolius*, revealed that the leaves possess a higher total flavonoid content than the fruits, suggesting that the leaves may serve as a more abundant source of phytochemicals [20]. Notably, the leaves of *R. parvifolius* were found to contain high concentrations of 2″-*O*-*trans*-*p*-coumaroyl astragalin and isoquercitrin [20], highlighting their potential therapeutic relevance.

In this study, chemical profiling of RPL was performed using LC-MS/MS and HPLC techniques. The extraction yielded 1.9 g of dried extract from 10 g of powdered material, representing a 19% yield.

**Figure 4:**
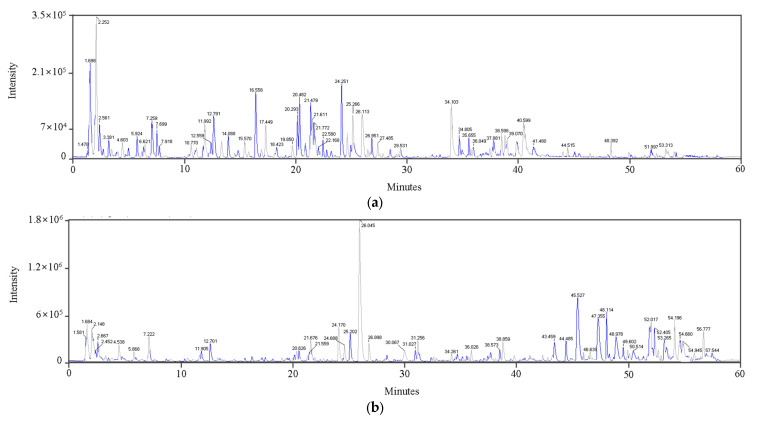
Total ion chromatograms of the RPL extract obtained by LC-ESI/MS analysis under (**a**) negative and (**b**) positive ionization modes.

LC-MS/MS analysis identified major flavonoids and phenolic compounds in RPL extract. Quantitative analysis focused on quercetin derivatives, ellagic acid, hirsutrin, tiliroside, and kaempferol derivatives. These compounds were selected due to their high abundance, clear chromatographic resolution, and availability of authentic standards. Minor or trace constituents were not quantified because of their low levels and the lack of commercially available standards (Figure 4, Table 2). These compounds are likely contributors to the observed anti-inflammatory effects, while minor components were not further analyzed. These assignments were supported by comparisons with reference spectra from the MoNA and NIST tandem mass spectral libraries, as well as an in-house spectral library.

**Figure 5:**
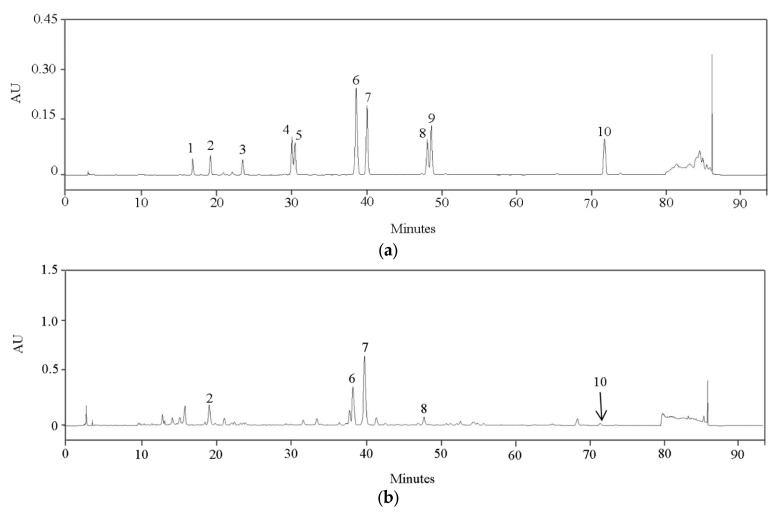
HPLC chromatograms of compounds **1**–**10** (**a**) and RPL extract (**b**). Compounds: chlorogenic acid (**1**), quercetin 3,7-diglucoside (**2**), epicatechin (**3**), quercetin 3,4′-diglucoside (**4**), kaempferol 3,4′-di-*O*-glucoside (**5**), ellagic acid (**6**), hirsutrin (**7**), kaempferol 3-*O*-glucuronide (**8**), quercetin-3-glucose-6″-acetate (**9**), and tiliroside (**10**).

The HPLC chromatograms of compounds **1**–**10** and the RPL extract are presented in Figure 5. The identified compounds were quercetin 3,7-diglucoside (**2**), ellagic acid (**6**), hirsutrin (**7**), kaempferol 3-*O*-glucuronide (**8**), and tiliroside (**10**). Quantitative analysis indicated that quercetin 3,7-diglucoside (**2**) was the most abundant component in the extract, followed by hirsutrin (**7**). Moderate levels of ellagic acid (**6**) and kaempferol 3-*O*-glucuronide (**8**) were also detected, whereas tiliroside (**10**) was present below the quantification limit. The total concentration of these compounds in the RPL extract was determined to be 19.36 mg per gram of dry weight (DW) (Table 3). The high abundance of quercetin 3,7-diglucoside (**2**) and the presence of hirsutrin (**7**) in the RPL extract may contribute to its observed anti-inflammatory effects at the cellular level.

A comparison with other *Rubus* species reveals that the major flavonoids in RPL, including quercetin 3,7-diglucoside and kaempferol 3-*O*-glucuronide, predominantly exist in glycosylated forms, whereas quercetin and kaempferol aglycones are more common in other *Rubus* species. Furthermore, the total content of these bioactive flavonoids in RPLs is higher than that reported for related *Rubus* species [9]. These findings highlight the distinctive phytochemical profile of RPL, suggesting that it may serve as a more potent source of anti-inflammatory compounds for dermatological applications.

Quercetin 3,7-diglucoside (**2**), hirsutrin (**7**), and ellagic acid (**6**), previously quantified as the major components in RPL extract, are likely contributors to the observed anti-inflammatory effects. The anti-inflammatory effects may be primarily attributed to these individual compounds. However, the crude extract contains multiple bioactive constituents whose combined presence may contribute to the overall activity. This raises the possibility of synergistic or additive interactions among the compounds, which could enhance the observed cellular effects. Future studies are warranted to evaluate the combined effects of major constituents to better understand potential synergistic mechanisms. These findings suggest that the anti-inflammatory activity observed in vitro is likely driven by the combined contributions of these dominant compounds, which have been independently reported to modulate AD-related inflammatory pathways. However, their individual contributions were not experimentally determined in this study, and future work should evaluate isolated compounds to clarify their specific roles. Although compounds such as tiliroside were present only in trace amounts and were not quantitatively analyzed, their documented anti-inflammatory activities suggest that even minor constituents could contribute to the overall cellular effects of the crude RPL extract [21]. Therefore, the observed anti-inflammatory activity may result from synergistic or additive interactions among both major and minor flavonoids, warranting further studies on isolated and combined constituents.

Quercetin and its glycosylated derivatives, such as quercetin 3,7-diglucoside (**2**), have been shown to alleviate inflammatory responses associated with AD by modulating oxidative stress and key signaling pathways, including MAPK [22]. In HaCaT cells, quercetin has been reported to reduce the expression of IL-6 and IL-8, thereby attenuating AD-like inflammation [23]. Ellagic acid (**6**), which is abundantly present in RPL, modulates inflammatory pathways such as MAPK and STAT, supporting its potential role in the anti-inflammatory effects observed in AD models [15,24]. In murine studies, ellagic acid has demonstrated efficacy in alleviating AD symptoms [24]. Hirsutrin (**7**), a glycosylated quercetin derivative, has been isolated from medicinal plants such as *Orthosiphon aristatus* [25]. This compound exhibits anti-inflammatory, anti-pruritic, and anti-allergic activities by suppressing mediators such as MIP-2 and various chemokines [25,26]. Hirsutrin (**7**) inhibits NO, PGE2, IL-1β, and IL-6 via MAPK, Akt, and NF-κB downregulation in LPS-stimulated microglia. A similar mechanism may occur in keratinocytes [18]. Additionally, kaempferol and its metabolite, kaempferol 3-*O*-glucuronide (**8**), have demonstrated anti-inflammatory and immunomodulatory activities relevant to AD, including suppression of Th2-mediated responses and improvement of clinical markers in experimental models [27,28,29]. Although we propose plausible pathway modulation consistent with the literature and the compounds identified, these signaling pathways (e.g., NF-κB, MAPK) were not directly investigated in the present study. Furthermore, the effects of RPL were evaluated only in vitro, without in vivo confirmation. Future studies should experimentally validate these mechanistic pathways and assess the physiological relevance of RPL in animal models to strengthen the translational significance of our findings.

Even though major flavonoids in RPL extract were identified, the individual contributions of these compounds to the observed anti-inflammatory activity remain unclear. Future studies should evaluate the bioactivity of isolated constituents to better understand the mechanisms involved.

## 4. Conclusions

The findings of this study support previous reports highlighting the rich diversity of bioactive compounds in RPL, further reinforcing its potential as a reliable and sustainable source of phytochemicals. Flavonoids were the predominant compounds and are known to modulate inflammatory pathways and immune responses. RPL extract effectively suppressed key cytokines such as IL-6 in keratinocytes, linking its phytochemical profile to anti-inflammatory activity relevant to AD. The presence of quercetin glycosides, hirsutrin, ellagic acid, and kaempferol 3-*O*-glucuronide highlights the multi-targeted mechanisms underlying the anti-inflammatory effects of RPL. These flavonoid-rich constituents may offer a promising natural alternative for managing AD by addressing multiple aspects of the inflammatory cascade. Overall, RPL extract exhibits considerable potential as a plant-derived therapeutic agent for treating inflammatory skin disorders, particularly chronic conditions such as AD. This study advances the field by linking phytochemical composition with anti-inflammatory efficacy, offering a basis for developing novel plant-based interventions for AD.

## Figures and Tables

**Table 1:** Gene-specific primer sequences used for qRT-PCR analysis.

Target	Primer Sequence (5′ to 3′)
*Glyceraldehyde-3-phosphate dehydrogenase (GAPDH)*	GTCTTCACCACCATGGAGAA AGGAGGCATTGCTGATGAT
*Monocyte chemoattractant protein 1 (MCP-1)*	TCAAACTGAAGCTCGCACTCT GGCATTGATTGCATCTGGC
*Normal T cell expressed and secreted (RANTES)*	CATATTCCTCGGACACCACACCCT ACTCCTGACCTCAAGTGATCCACC
*Thymus and activation-regulated chemokine (TARC)*	GCAGCTCGAGGGACCAATGT TTGGGGTCCGAACAGATGGC
*Macrophage-derived chemokine (MDC)*	AGGACAGAGCATGGCTCGCCTACAG TAATGGCAGGGAGGTAGGGCTCCTG
*Cutaneous T Cell-Attracting Chemokine (CTACK)*	CACTGCCTGCTGTACTCAGCTCTA CTTCAGCCCATTTTCCTTAGCATC
*Interleukin 6 (IL-6)*	GTGTGAAAGCAGCAAAGAGGC CTGGAGGTACTCTAGGTATAC

**Table 2:** Tentative structural identification of compounds in RPL extract based on LC-MS/MS analysis under positive and negative ionization modes.

t_R_ (min)	MW	Tentative Identification
12.30	578.1	Procyanidin B_2_ ^1^
13.54	626.1	Quercetin 3,7-diglucoside ^1,2^
14.20	290.1	Epicatechin ^1,2^
15.45	610.2	Kaempferol 3,4′-di-*O*-glucoside ^1,2^
19.83	610.2	Rutin ^1^
19.96	464.1	Hyperoside ^2^
20.04	302.0	Ellagic acid ^1^
20.07	478.1	Quercetin glucuronide ^2^
20.37	464.1	Hirsutrin ^1,2^
21.21	506.1	Quercetin 3-*O*-glucose-6″-acetate ^1^
21.65	462.1	Kaempferol 3-*O*-glucuronide ^1,2^
21.70	448.1	Maritimein ^1,2^
23.95	368.1	5-*O*-Caffeoylquinic acid methyl ester ^1,2^
24.35	666.4	1-*O*-(20,24-Epoxy-1,3,25-trihydroxy-28-oxo-9,19-cyclolanostan-28-yl)-hexopyranose ^1^
24.88	302.0	Quercetin ^1,2^
25.21	594.1	Tiliroside ^1,2^
37.82	504.3	Madecassic acid ^1,2^
45.56	456.4	Oleanolic acid ^2^

^1^ Negative ion mode. ^2^ Positive ion mode.

**Table 3:** Content of compounds **1**–**10** identified in the RPL extract.

Sample	Standard Compound (mg/g DW)										
	1	2	3	4	5	6	7	8	9	10	Total
RPL	ND	12.73 ± 0.14	ND	ND	ND	1.58 ± 0.01	4.74 ± 0.04	0.31 ± 0.01	ND	trace	19.36

ND: not detected, Compounds: chlorogenic acid (**1**), quercetin 3,7-diglucoside (**2**), epicatechin (**3**), quercetin 3,4′-diglucoside (**4**), kaempferol 3,4′-di-*O*-glucoside (**5**), ellagic acid (**6**), hirsutrin (**7**), kaempferol 3-*O*-glucuronide (**8**), quercetin-3-glucose-6″-acetate (**9**), and tiliroside (**10**).

## Data Availability

All original data presented in this study are included in the article. Further inquiries can be directed to the corresponding author.

## Abbreviations

The following abbreviations are used in this manuscript:

AD	Atopic dermatitis
IL-6	Interleukin-6
IL-8	Interleukin-8
MCP-1	Monocyte chemoattractant protein-1
ROS	Reactive oxygen species
TNF-α	Tumor necrosis factor-α
IFN-γ	Interferon gamma
LC-MS/MS	Liquid chromatography-tandem mass spectrometry
HPLC	High-performance liquid chromatography
RPL	*Rubus parvifolius* leaf
DMEM	Dulbecco’s modified Eagle’s medium
MTT	3-(4,5-Dimethylthiazol-2-yl)-2,5-diphenyltetrazolium bromide
FBS	Fetal bovine serum
P/S	Penicillin-streptomycin
DMSO	Dimethyl sulfoxide
ACN	Acetonitrile
ELISA	Enzyme-linked immunosorbent assay
qRT-PCR	Quantitative reverse transcription PCR
TI	TNF-α and IFN-γ
PBS	Phosphate-buffered saline
RANTES	Regulated upon activation normal T cell expressed and secreted
TARC	Thymus and activation-regulated chemokine
MDC	Macrophage-derived chemokine
CTACK	Cutaneous T cell-attracting chemokine
GAPDH	Glyceraldehyde-3-phosphate dehydrogenase
DW	Dry weight
